# Supplementary material for: Biomimetic Superhydrophobic Films with an Extremely Low Roll-Off Angle Modified by F16CuPc via Two-Step Fabrication
Source: Nanomaterials (Basel). 2022 Mar 14;12(6):953. doi: 10.3390/nano12060953 (PMC8953802; doi:10.3390/nano12060953)
Supplement: Supplementary file 1 [file nanomaterials-12-00953-s001.zip › nanomaterials-1613755-supplementary.pdf]

Supplementary Materials

Biomimetic Superhydrophobic Films with an Extremely Low Roll-Off Angle Modified by $F_{16}CuPc$ via Two-Step Fabrication

Pengchao Zhou †, Tengda Hu †, Yachen Xu, Xiang Li, Wei Shi, Yang Lin *, Tao Xu and Bin Wei *

School of Mechatronic Engineering and Automation, Shanghai University, Shanghai 200444, China; pczhou@shu.edu.cn (P.Z.); hutengda@shu.edu.cn (T.H.); xuyachen1985311@163.com (Y.X.); 2575154029@shu.edu.cn (X.L.); shiwei@shu.edu.cn (W.S.); xtld@shu.edu.cn (T.X.)

* Correspondence: ylin@i.shu.edu.cn (Y.L.); bwei@shu.edu.cn (B.W.)

† These authors contributed equally to this work.

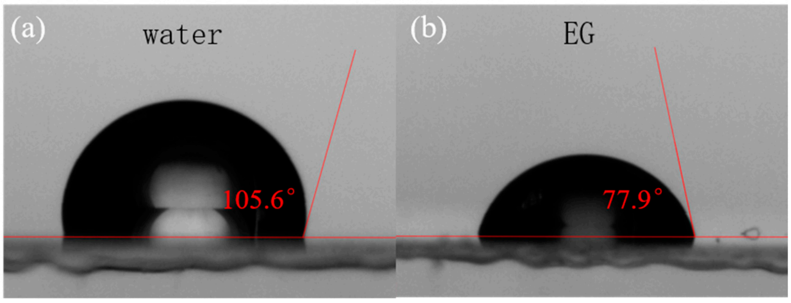

Figure S1. The wettability of the pure $F_{16}CuPc$ film. (a) the WCA and (b) the EGCAon the surface of pure $F_{16}CuPc$.

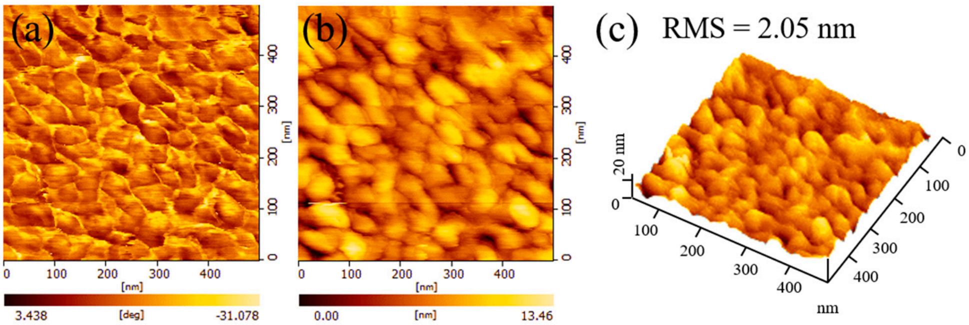

Figure S2. The AFM images of the pure $F_{16}CuPC$ film. (a) the topography image, (b) the phase images, and (c) the 3d images of the pure $F_{16}CuPC$ film.

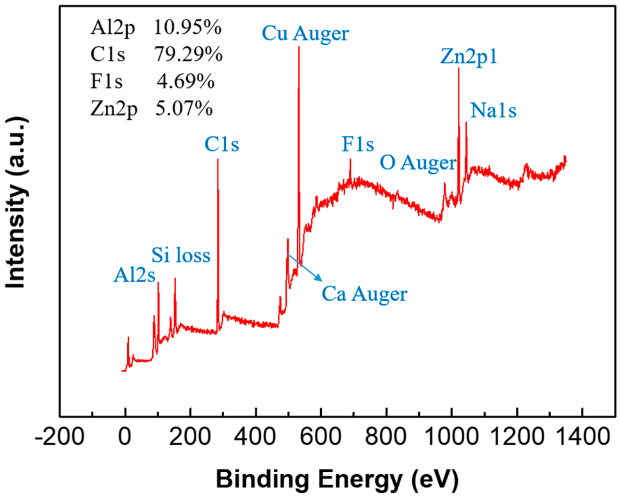

Figure S3. The XPS spectrum of the $F_{16}CuPc$-modified biomimetic superhydrophobic film.

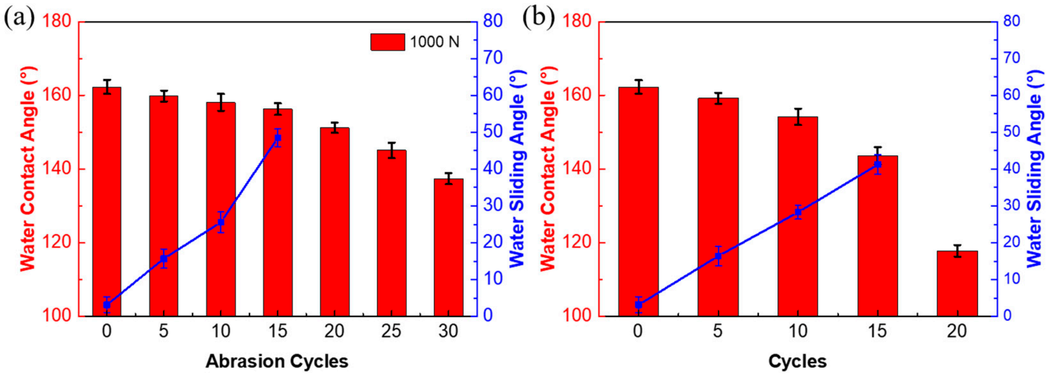

Figure S4. The changes of WCA and WRA of the biomimetic superhydrophobic film without modification after (**a**) the wear test with 1000 N weight and (**b**) tape stripping experiment.

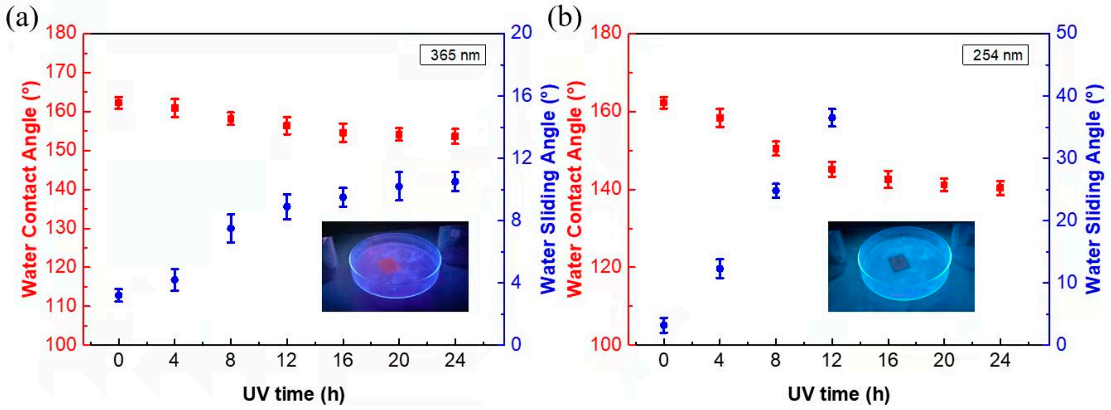

Figure S5. The changes of WCA and WRA of the stacked substrate after UV irradiation with (**a**) 365 nm and (**b**) 254 nm UV lamp.

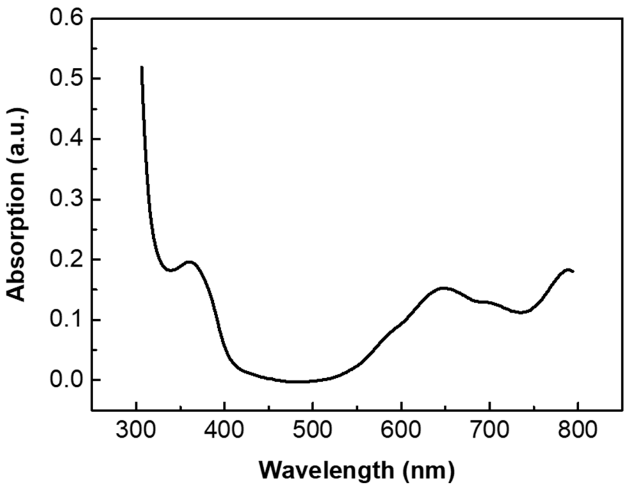

Figure S6. The absorption spectrum of the pure $F_{16}CuPC$ film.

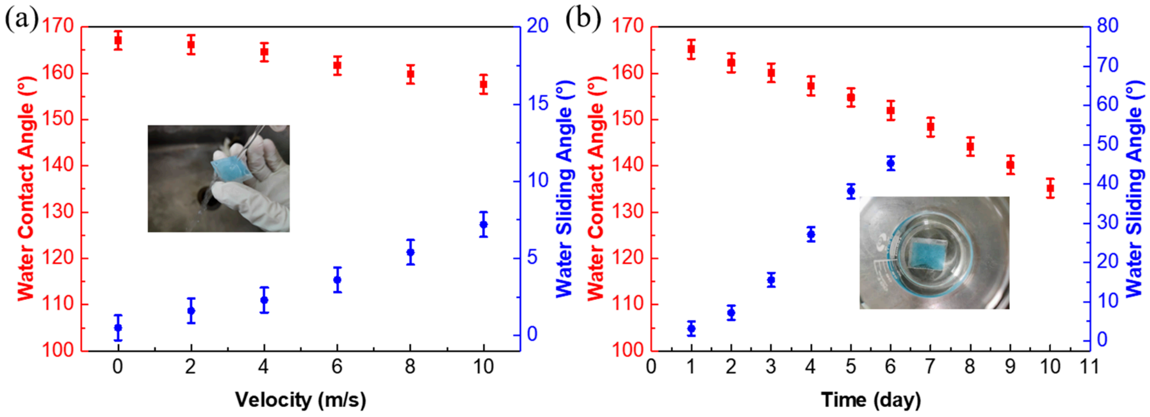

Figure S7. Changes of the WCA and WRA of the $F_{16}CuPc$-modified biomimetic superhydrophobic film under the (**a**) water impact and (**b**) soaking.

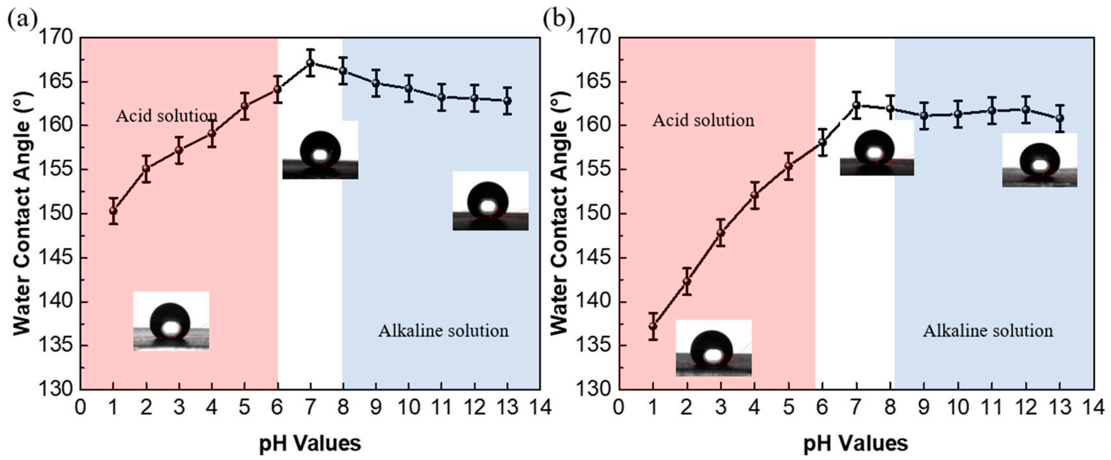

Figure S8. The changes of the WCA of the droplets with different pH values for (**a**) the $F_{16}CuPc$-modified biomimetic superhydrophobic surface and (**b**) the surface without modification.

Preparation process of MS

First ground the aluminum isopropoxide into powder form in an agate mortar and added 8.3 g to the beaker. Then added 3.2 g NaOH and 40 g deionized water to the beaker. The aluminum sol was formed by stirring at 60 °C for approximately 1 h. After that, added 8.2 g 30 % alkaline silica sol, 1.8 g sodium hydroxide, and 20 g deionized water to the beaker and stir for 1 h to make it homogeneous. The synthesized silica sol was slowly added dropwise to the aluminum sol at room temperature (25 ℃) stirred for 1 h. Then 0.2 g 25 % TMAOH template agent was added and stirred for 12 h. The above solution was added to the hydrothermal reactor at 100 ℃ for 2 h. After cooling, the aqueous sample was separated by centrifugation in deionized water, and then dried at 110 ℃ under vacuum conditions to obtain $Na_{12}Al_{12}Si_{12}O_{48}·27H_2O$. The $Na_{12}Al_{12}Si_{12}O_{48}·27H_2O$ crystals were ion exchanged in 0.5 mol/L $CaCl_2$ solution at 55 ℃ in a 1:20 solid-liquid ratio for 1 h. The crystals were washed by filtration to neutral and dried. Finally, multi-stage pore MS ($3/4CaO1/4Na_2OAl_2O_3·2SiO_2·4.5H_2O$) with mesoporous structures were obtained by activation in a muffle furnace at 450 ℃.
